# Expansion of Cyclophyllidea Biodiversity in Rodents of Qinghai-Tibet Plateau and the “Out of Qinghai-Tibet Plateau” Hypothesis of Cyclophyllideans

**DOI:** 10.3389/fmicb.2022.747484

**Published:** 2022-02-08

**Authors:** Yao-Dong Wu, Guo-Dong Dai, Li Li, D. Timothy J. Littlewood, John Asekhaen Ohiolei, Lin-Sheng Zhang, Ai-Min Guo, Yan-Tao Wu, Xing-Wei Ni, Nigus Abebe Shumuye, Wen-Hui Li, Nian-Zhang Zhang, Bao-Quan Fu, Yong Fu, Hong-Bin Yan, Wan-Zhong Jia

**Affiliations:** ^1^State Key Laboratory of Veterinary Etiological Biology, National Professional Laboratory for Animal Echinococcosis, Key Laboratory of Veterinary Parasitology of Gansu Province, Lanzhou Veterinary Research Institute, Chinese Academy of Agricultural Sciences, Lanzhou, China; ^2^Department of Life Sciences, Natural History Museum, London, United Kingdom; ^3^London Centre for Neglected Tropical Disease Research, London, United Kingdom; ^4^Guizhou Provincial Center for Animal Disease Control and Prevention, Guiyang, China; ^5^State Key Laboratory of Plateau Ecology and Agriculture, Qinghai Academy of Animal Science and Veterinary Medicine, Qinghai University, Xining, China

**Keywords:** Cyclophyllidea, phylogeny, species differentiation, biogeography, Qinghai-Tibet Plateau, rodents

## Abstract

The Cyclophyllidea comprises the most species-rich order of tapeworms (Platyhelminthes, Cestoda) and includes species with some of the most severe health impact on wildlife, livestock, and humans. We collected seven Cyclophyllidea specimens from rodents in Qinghai-Tibet Plateau (QTP) and its surrounding mountain systems, of which four specimens in QTP were unsequenced, representing “putative new species.” Their complete mitochondrial (*mt*) genomes were sequenced and annotated. Phylogenetic reconstruction of partial 28S rDNA, *cox*1 and *nad*1 datasets provided high bootstrap frequency support for the categorization of three “putative new species,” assigning each, respectively, to the genera *Mesocestoides*, *Paranoplocephala*, and *Mosgovoyia*, and revealing that some species and families in these three datasets, which contain 291 species from nine families, may require taxonomic revision. The partial 18S rDNA phylogeny of 29 species from Taeniidae provided high bootstrap frequency support for the categorization of the “putative new species” in the genus *Hydatigera*. Combined with the current investigation, the other three known Taeniidae species found in this study were *Taenia caixuepengi*, *T. crassiceps*, and *Versteria mustelae* and may be widely distributed in western China. Estimates of divergence time based on *cox*1 + *nad*1 fragment and *mt* protein-coding genes (PCGs) showed that the differentiation rate of Cyclophyllidea species was strongly associated with the rate of change in the biogeographic scenarios, likely caused by the uplift of the QTP; i.e., species differentiation of Cyclophyllidea might be driven by host-parasite co-evolution caused by the uplift of QTP. We propose an “out of QTP” hypothesis for the radiation of these cyclophyllidean tapeworms.

## Introduction

Cestoda is a class of parasitic worms in the flatworm phylum Platyhelminthes that parasitize the intestines of all major groups of vertebrates, including fish (Kuchta et al., 2020), reptiles (Yudhana et al., 2019), birds (Okulewicz, 2014), and mammals (Carlson et al., 2020), and cause severe, mild or no symptoms of infection. Their larvae usually achieve development in one or two intermediate hosts (Kuchta et al., 2020) and can cause severe symptoms and even death in animals and humans; species of *Taenia* and *Echinococcus* cause the highest health and economic impact.

To date, there are approximately 4,800 described species in the class Cestoda, belonging to 833 genera and 19 orders, of which the Cyclophyllidea is the most species-rich order in the class, with more than 3,100 valid species, distributed among 16 families (Sharma et al., 2016; Caira and Jensen, 2017; Kuchta et al., 2020). Each free-living metazoan species is considered to harbor at least one protozoan or metazoan parasite species (Poulin and Morand, 2004) with which they interact and usually co-evolve (Ebert and Fields, 2020). Parasites are probably one of the largest groups of undescribed organisms, as most are cryptic whether in their parasitic or free-living forms (Dobson et al., 2008; Larsen et al., 2017; Okamura et al., 2018; Carlson et al., 2020). It is estimated that there are a total of about 100,000–350,000 helminth species in vertebrates around the world, of which 85–95% are undiscovered or recorded to science (Carlson et al., 2020).

Many of the world’s biodiversity hotspots are located in large mountain systems, and their role in the evolutionary diversification of organisms is manifold (Hoorn et al., 2018; Rahbek et al., 2019a,b). The Qinghai-Tibet Plateau (QTP) and its surrounding mountain systems of the Eurasian continent, have yielded arguably the biggest and probably the most biologically diverse area of montane species (Päckert et al., 2020). In the past decade, several new Taeniidae species have been described from wild rodents on the QTP (Xiao et al., 2005; Wu et al., 2021). In terms of species diversity and sheer population sizes, rodents are perhaps the most important intermediate and definitive hosts of tapeworms, and are the most widely distributed and diverse group of mammals; about 43% of all species (Singla et al., 2008; Wu et al., 2018). Changes in climate and vegetation caused by the uplift of the QTP may have promoted local adaptations such as the evolution of cold- and hypoxic-tolerant rodent species, which in turn may have led to the co-evolution and radiation of their parasites (Wang et al., 2018; Wu et al., 2021). We hypothesize that there may be many undiscovered tapeworm species parasitizing the rodents in the QTP. Although the number of tapeworm species has been underestimated in general, some practices may lead to the erroneous proposal of new species (Carlson et al., 2020; Kuchta et al., 2020).

Morphological distinctions have been used for the description of many tapeworms, however, the homoplasy in morphology poses quite a challenge to infer their evolutionary lineage (Scholz et al., 2021), where morphological features may be significantly affected by different intermediate host sources (Lymbery, 1998). Many undescribed species may be genetically different but morphologically indistinguishable. The inability to distinguish such cryptic species affects accurate assessment of host range and estimates of total diversity (Carlson et al., 2020). The 28S and 18S nuclear ribosomal RNA genes (rDNA) are relatively conserved within species and are often used to differentiate different species (Wickström et al., 2005; Nakao et al., 2013; Scholz et al., 2021). The mitochondrial (*mt*) genome is largely haploid and uniparentally inherited, so their effective population size is four times smaller than that of the nuclear genome (Toews and Brelsford, 2012). Since the process of lineage sorting is inversely proportional to the effective population size, this means mitochondrial (*mt*) genomes will complete this process faster than nuclear genomes (Toews and Brelsford, 2012). Thus, the genetic nature of a *mt* genome makes it likely more sensitive than any single nuclear marker to distinguish closely related species and study their phylogenetic relationships (Lee et al., 2007).

Therefore, in this study, we sampled parasites in rodents from the QTP and its surrounding areas. In total, seven Cyclophyllidea specimens were collected and characterized using molecular tools. By comparing new (mitochondrial, nuclear 28SrDNA, 18S rDNA) with published homologs (GenBank) we found four isolates to be markedly different in DNA sequence thus likely representing ‘‘putative new species.’’ The complete *mt* genomes of these unknown taxa were sequenced and annotated, and their taxonomic status was analyzed and verified through phylogenetic reconstruction of five datasets containing a total of 320 species from 10 families. By combining evolutionary divergence time analyses of *mt* genes of classified cyclophyllideans in NCBI Taxonomy Database^1^ and the paleogeography of QTP, we thus speculate an “out of QTP” theory for cyclophyllideans.

## Materials and Methods

### Sample Collection

Rodents were live-trapped in meadows in Tibet, Qinghai, Sichuan, Gansu and Xinjiang province or autonomous region of China in 2013 and from 2018 to 2020. Rodents were euthanized and dissected according to the Ethics Statement mentioned below, cysticerci and host livers were collected from the enterocoelia and thorax, and adults were extracted from the intestines. Detailed sample collection data and host identities are described in Supplementary Table 1. After detaching the lesions, parasite specimens and host livers were kept in 75% (v/v) ethanol for molecular identification.

### DNA Isolation, Amplification, and Sequencing

DNA samples of hosts and parasites were extracted using Blood and Tissue Kit (Cat. No. 69504, Qiagen, Germany) as instructed by the manufacturer, and were amplified and sequenced for identification by conserved primers of *cyt*b gene of small mammals (Fan et al., 2011) and *cox*1 gene of tapeworm (Bowles et al., 1992), respectively. By means of highly similar BLAST search in the nucleotide collection (nr/nt) database,^2^ four of the Cyclophyllidea specimens with less than 95% identity of the most similar *cox*1 sequence were identified as putatively unknown species (Xiao et al., 2005). The *mt* genomes of four of the putatively unknown specimens were sequenced and assembled according to the following procedure: firstly, the DNA of the four species was amplified and sequenced using primers published in Wu et al. (2021); missing fragments were amplified with newly designed primers using the program Oligo 6.0 with the method described in Wu et al. (2021), until entire circular *mt*DNAs were amplified and sequenced. A list of primers for each species can be found in Supplementary Table 2. The 18S and 28S rDNA fragments of these four species were also amplified and sequenced with conserved primers (Littlewood et al., 2000; Yan et al., 2013) for further species identification and phylogenetic analyses. Primers were synthesized by Tsingke Biotechnology (Xi’an, China). Standard 25 μl PCR protocol was used to amplify the DNA fragments. The PCR products were purified and sequenced according to protocols in Wu et al. (2021). SeqMan software was used to assemble the *cyt*b gene sequences, 18S and 28S rDNA partial sequences and *mt* genomes (see Supplementary Table 3 for GenBank accession nos.).

### Mitochondrial Genome Annotation

**FIGURE 1:**
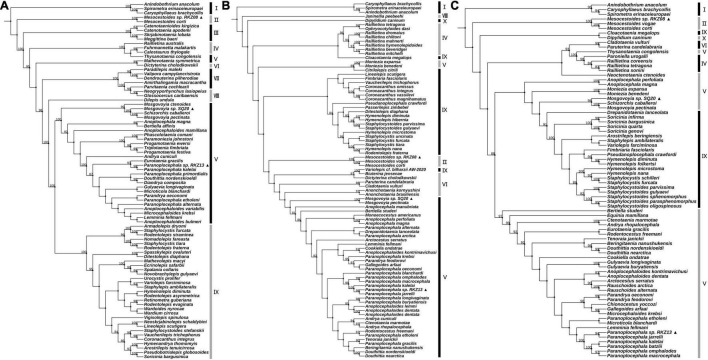
Maximum likelihood analyses of the four “putative new species” with other Cyclophyllidea species, except for Taeniidae, based on simplified 28S rDNA **(A)**, *cox*1 **(B)** and *nad*1 **(C)** fragments. Alternating black and gray bands are used to classify different genera in the Taxonomy of NCBI, The Roman numerals to the right of the bands represent outgroup and different genus names (i.e., I: outgroup, II: Mesocestoididae, III: Catenotaeniidae, IV: Dipylidiidae, V: Anoplocephalidae, VI: Paruterinidae, VII: Gryporhynchidae, VIII: Dilepididae, IX: Hymenolepididae, X: Dipylidiidae). The ▲ after the species name represents “putative new species”. Bootstrap frequency support values are stated only for nodes where > 80%.

**FIGURE 2:**
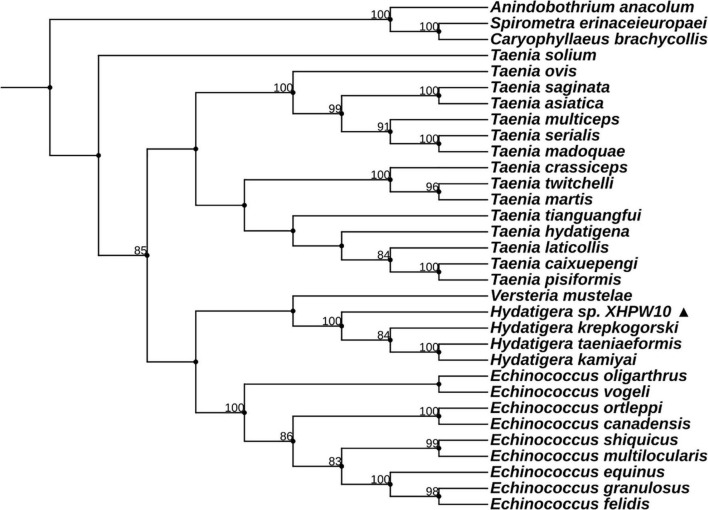
Maximum likelihood analysis of 18S rDNA of family Taeniidae. The ▲ after the species name represents “putative new species.” The outgroup is the same as Figure 1. Bootstrap frequency support values are stated only for nodes where > 80%.

The four new *mt*DNAs were annotated preliminarily by Geseq^3^ with the reference of the most closely related species (*Mesocestoides corti*, *Anoplocephala magna*, *Hydatigera krepkogorski*, and *Moniezia expansa*) identified by the phylogenetic analyses in Figures 1, 2, whose *mt* genome annotations are available in GenBank. Putative tRNA genes were verified using ARWEN^4^ using default parameters (Laslett and Canbäck, 2008). The positions of their open reading frames (ORF) and rRNA genes were further checked and modified using SnapGene (v3.2.1) based on alignments with the reference of the most closely related species mentioned above. SnapGene (v3.2.1) was used to translate the protein-coding genes (PCGs) into their amino acid sequence with echinoderm/flatworm mitochondrial code (NCBI translation table 9) and to illustrate the annotated *mt* genomes.

### Phylogenetic Analyses and Sequence Identity

As ingroups for phylogenetic reconstruction, we combined *mt* genes and 18S or 28S rDNA sequenced in this study with those of other classified cyclophyllideans available in GenBank. Three species (*Caryophyllaeus brachycollis*, *Anindobothrium anacolum*, *Spirometra erinaceieuropaei*) of Eucestoda belonging to different orders were chosen as outgroups.

The 28S rDNA fragments of classified cyclophyllideans in NCBI Taxonomy Database are available for most families in GenBank except Taeniidae, while that of the 18S rDNA were only available for Taeniidae, so here the evolutionary trees of 28S and 18S rDNA fragments were constructed separately. Based on the results of the 28S rDNA phylogeny, a simplified 28S evolutionary tree was reconstructed without affecting the topological structure of the tree by removing the species in the same genus and clade. To complement and validate the inferred evolutionary relationship between Cyclophyllidea species, except the species of family Taeniidae, the phylogenies with *cox*1 and *nad*1 fragments were reconstructed.

In summary, 5 datasets containing 320 species in 10 families were assembled for phylogenetic analyses (see Supplementary Tables 4–8 for GenBank accession nos. and species, genera and families used in each dataset). Since the limited availability of data on different genes of the same species, we performed separate phylogenetic reconstruction for each dataset to cover more species. Datasets were aligned using MAFFT v7.487 with auto option (Katoh and Standley, 2013). The alignments were trimmed by using Trimal v1.2 under the automated1 option (Capella-Gutiérrez et al., 2009) to preserve the same sequence regions and exclude the ambiguously aligned sites. All phylogenetic trees were constructed with maximum likelihood (ML) inference using IQ-TREE v2.1.4 (Nguyen et al., 2015) with ultrafast bootstrap 1,000 replicates in Ubuntu 20.04.2 LTS operating system, where the best-fit models were automatically selected by ModelFinder (Supplementary Table 9; Kalyaanamoorthy et al., 2017) and the best number of threads were also selected under AUTO option (using the command line: iqtree -s alignment_file -m -MFP -bb 1000 -nt AUTO). All other parameters were set to their default values. The ML trees were then visualized on the IToL web server (Letunic and Bork, 2016).

The related species of the putatively unknown species were identified based on phylogenetic reconstruction, and the percentage identity of sequences in the 5 datasets between the putatively unknown species and their related species was calculated by BLAST (Supplementary Table 10).

### Analysis of Divergence Times

Species divergence times were calculated using BEAST v2.6.2 (Bouckaert et al., 2014) based on two datasets without shared species: all available *mt* PCGs dataset of 54 species (Supplementary Table 11) and *cox*1 + *nad*1 fragments dataset of other classified 54 species (Supplementary Table 12). These two datasets were aligned and trimmed with MAFFT v7.487 and Trimal v1.2 as described above, and were partitioned according to different genes. Model selection of partitions was identified by Partition Finder v2.1.1 (Lanfear et al., 2012) with the set of “linked” branch lengths, “beast” models, “aicc” model selection, and “greedy” search. Partition schemes and substitution models can be found in Supplementary Table 9. The Strict Clock model was chosen to ignore the rate differences between the branches in the mode and the gamma category count was set to 4. Other settings, such as substitution rate and shape, in the site model were evaluated in the analysis. The Calibrated Yule model (Heled and Drummond, 2015) was used as the tree prior, as it is a simple model of speciation that is generally appropriate when considering sequences from different species. Time calibration was calibrated with the divergence date between *T. saginata* and *T. asiatica* (∼1.14 Mya) and divergence date between *Schistosoma japonicum* and *S. mansoni* (∼56.10 Mya), which agreed with reported fossil evidence from shark coprolites and previously estimated dates based on *mt* genes (Wang et al., 2016). Posterior probability estimates were sampled every 1,000 iterations over a total of 10,000,000 iterations per MCMC run. Other options were run on their default values. Tracer (v1.7.1) was used to summarize posterior probabilities. Trees were annotated via TreeAnnotator (v2.1.2) using a maximum clade credibility tree and median heights settings with 10% burn-in. The number of divergence nodes in every 2 Mya was summarized based on the evolutionary divergence time trees of two datasets, respectively.

## Results

### Species Identification and Phylogenetic Relationships

The hosts of parasites were confirmed by BLAST searches (Supplementary Table 1; see Supplementary Table 3 for GenBank accession nos. of host *cyt*b gene). Two of the parasite species were cysticerci and adult worms, each retrieved from the abdominal cavity and intestinal tract of two vole hosts (*Neodon leucurus*) collected from the same pasture location near the Shigatse City of Tibet Autonomous Region (Supplementary Table 1). Phylogenetic analyses shown in Supplementary Figure 1 and Figures 1A–C confirmed that these two species likely belong to the genera *Mesocestoides* and *Paranoplocephala*, and were labeled as *Mesocestoides* sp. RKZ08 and *Paranoplocephala* sp. RKZ13, respectively. The cysticerci collected from the liver of plateau pika (*Ochotona curzoniae*) from Xietongmen county of Tibet and zokors (*Eospalax fontanierii*) from Xiahe county of Gansu province were confirmed to be the same species by alignment of *cox*1 and 18S rDNA segments, which was in the monophyletic group of the genus *Hydatigera* in the phylogenetic trees (Figure 2), and was marked as *Hydatigera* sp. XHPW10. The final species was an adult tapeworm collected from the intestine of plateau pikas from Shiqu county, Sichuan province, and occurred within the genus *Mosgovoyia* (Supplementary Figure 1 and Figures 1A–C), and was thus named *Mosgovoyia* sp. SQ20. These four species were identified as newly sequenced and “putative new species” as their *cox*1 and *nad*1 sequences having less than 95% identity with available related taxa (Supplementary Table 10; Xiao et al., 2005). The degree of differentiation of their *mt* genes was higher than that of the nuclear genes 18S and 28S rDNA (Supplementary Table 10), reflecting their differentiation likely results from the so-called deep mitochondrial divergence (DMD, Zhang et al., 2019). In addition, three known cysticerci of Taeniidae, *T. caixuepengi*, *T. crassiceps*, and *Versteria mustelae*, were also identified in the present study (see Supplementary Table 13 for their *cox*1 fragments).

### General Features of the Mitochondrial Genome of “Putative New Species”

**FIGURE 3:**
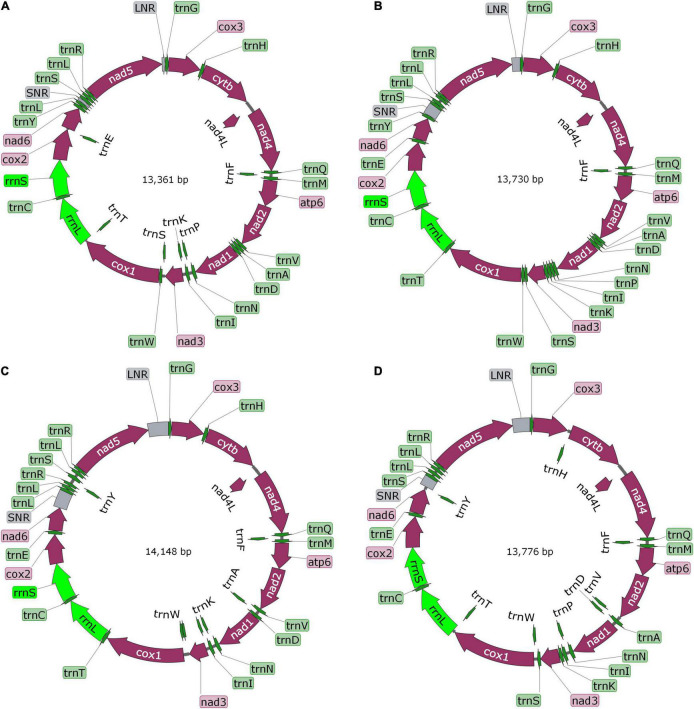
The diagram of complete mitochondrial genome of *Mesocestoides* sp. RKZ08 **(A)**, *Paranoplocephala* sp. RKZ13 **(B)**, *Hydatigera* sp. XHPW10 **(C)**, and *Mosgovoyia* sp. SQ20 **(D)**. The protein-encoding genes are depicted in plum, the tRNAs are depicted in green, the rRNAs are depicted in light green and the non-coding mitochondrial regions (NCRs including LNR and SNR) are depicted in gray. The inferred gene boundaries of them are shown in Table 1.

The complete *mt* genomes of the four “putative new species” were 13,361 bp (GenBank ID: MW808979), 13,730 bp (GenBank ID: MW808980), 14,148 bp (GenBank ID: MW808981), and 13,776 bp (GenBank ID: MW808982) in length. Each of them contains two rRNA genes (*rrn*S and *rrn*L) and 12 protein-encoding genes (*atp*6, *cyt*b, *nad*4L, *cox*1-3, and *nad*1-6), which are arrayed in the typical order of *mt* genomes of cestodes. They each contain the 22 typical tRNA genes set of cestodes, and share a common set of anticodons. The order of tRNA genes is roughly the same, except between *nad*6 and *nad*5 genes. Species *Paranoplocephala* sp. RKZ13 had a repeat sequence of *trn*L and *trn*R in the highly variable region between *nad*6 and *nad*5, suggesting that it had an insertion sequence in this region that made its *mt* genome longer than the other three species (Figure 3 and Table 1).

**TABLE 1:** The list of mitochondrial genome annotation for four “putative new species”.

Genes	Positions of nucleotide sequences (bp)				Initiation and termination codons				Anticodons
	RKZ08	RKZ13	XHPW10	SQ20	RKZ08	RKZ13	XHPW10	SQ20	
*trn*G	1–67	1–62	1–67	1–63					TCC
*cox*3	73–717	68–712	70–714	65–715	ATG/TAA	ATG/TAA	ATG/TAA	ATG/TAG	
*trn*H	726–794	713–783	716–784	709–778					GTG
*cyt*b	798–1892	787–1881	787–1,854	782–1876	ATG/TAG	GTG/TAG	ATG/TAG	GTG/TAG	
*nad*4L	1,895–2,155	1,884–2,144	1,877–2,137	1,892–2,152	GTG/TAG	ATG/TAA	GTG/TAG	ATG/TAA	
*nad*4	2,116–3,372	2,111–3,358	2,104–3,354	2,113–3,366	GTG/TAA	GTG/TAG	GTG/TAA	GTG/TAG	
*trn*Q	3,373–3,435	3,359–3,426	3,355–3,417	3,367–3,428					TTG
*trn*F	3,434–3,499	3,425–3,488	3,415–3,479	3,427–3,490					GAA
*trn*M	3,495–3,562	3,485–3,551	3,475–3,542	3,489–3,554					CAT
*atp*6	3,568–4,080	3,557–4,063	3,551–4,069	3,558–4,073	ATG/TAA	ATG/TAA	ATG/TAG	ATG/TAG	
*nad*2	4,103–4,978	4,073–4,948	4,076–4,966	4,083–4,964	ATG/TAG	ATG/TAG	ATG/TAG	ATG/TAA	
*trn*V	4,983–5,047	4,951–5,014	4,967–5,030	4,978–5,041					TAC
*trn*A	5,048–5,115	5,014–5,077	5,036–5,103	5,040–5,106					TGC
*trn*D	5,121–5,187	5,082–5,142	5,108–5,171	5,106–5,166					GTC
*nad*1	5,192–6,079	5,145–6,035	5,176–6,069	5,170–6,060	ATG/TAA	ATG/TAG	ATG/TAG	ATG/TAG	
*trn*N	6,095–6,160	6,041–6,109	6,070–6,137	6,066–6,131					GTT
*trn*P	6,163–6,226	6,117–6,180	6,145–6,207	6,137–6,200					TGG
*trn*I	6,227–6,290	6,180–6,245	6,206–6,269	6,200–6,264					GAT
*trn*K	6,294–6,358	6,244–6,308	6,274–6,336	6,275–6,337					CTT
*nad*3	6,362–6,709	6,310–6,657	6,334–6,681	6,342–6,689	ATG/TAA	ATG/TAG	GTG/TAG	ATG/TAA	
*trn*S	6,718–6,776	6,656–6,714	6,680–6,738	6,692–6,751					GCT
*trn*W	6,780–6,845	6,715–6,776	6,746–6,809	6,756–6,818					TCA
*cox*1	6,851–8,449	6,774–8,342	6,810–8,462	6,816–8,408	ATG/TAA	ATG/TAG	ATG/TAG	ATG/TAA	
*trn*T	8,456–8,518	8,365–8,429	8,427–8,490	8,395–8,458					TGT
*rrn*L	8,519–9,488	8,430–9,393	8,491–9,452	8,459–9,431					
*trn*C	9,489–9,554	9,394–9,450	9,453–9,511	9,432–9,496					GCA
*rrn*S	9,555–10,282	9,451–10,187	9,512–10,232	9,497–10,232					
*cox*2	10,283–10,861	10,188–10,760	10,233–10,859	10,233–10,808	ATG/TAA	ATG/TAA	ATG/TAA	ATG/TAA	
*trn*E	10,864–10,932	10,771–10,837	10,816–10,883	10,813–10,877					TTC
*nad*6	10,933–11,394	10,842–11,300	10,884–11,336	10,881–11,348	ATG/TAG	GTG/TAG	GTG/TAG	ATG/TAA	
*trn*Y	11,403–11,467	11,862–11,928	11,339–11,402	11,340–11,403					GTA
SNR	11,468–11,689	11,301–11,632	11,472–11,526	11,404–11,585					
*trn*L	11,757–11,824	11,633–11,698	11,410–11,471	11,667–11,728					TAG
*trn*L*		11,705–11,768							CAA
*trn*R*		11,782–11,838							ACG
*trn*S	11,690–11,755	11,927–11,999	11,527–11,593	11,586–11,647					TGA
*trn*L	11,863–11,925	12,046–12,111	11,602–11,663	11,751–11,813					TAA
*trn*R	11,926–11,979	12,124–12,180	11,674–11,731	11,830–11,888					ACG
*nad*5	11,984–13,564	12,181–13,752	11,735–13,297	11,889–13,457	ATG/TAA	ATG/TAA	ATG/TAG	ATG/TAA	
LNR	13,565–13,730	13,753–14,148	13,298–13,361	13,458–13,776					

*Stands for the gene in repeat region.

Flatworms use a unique set of *mt* code for protein translation (Nakao et al., 2000; Telford et al., 2000). In addition to ATG, GTG was also used as an initiation codon in a small fraction of coding genes in their *mt* genomes. For the termination codon, all species used only TAA and TAG; TGA was not identified as a termination codon (Table 1).

### Divergence Times Analysis

**FIGURE 4:**
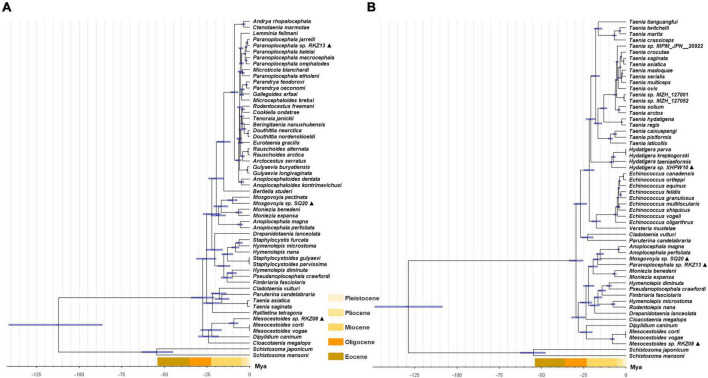
Divergence time construction of concatenated *cox*1 + *nad*1 gene **(A)** and 12 *mt* PCGs **(B)** of Cyclophyllidea species. The ▲ after the species name represents “putative new species”. The blue bar represents interval of 95% highest probability density. The time scale bars in different colors shows the extent of the Eocene, Oligocene, Miocene, Pliocene, and Pleistocene period.

**FIGURE 5:**
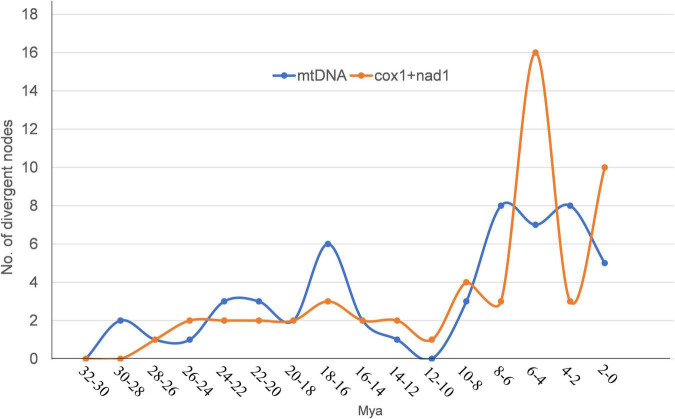
The number of divergent nodes in every 2 Mya over time based on the divergence time trees of Figure 4. The orange curve represents the change of divergence nodes number based on Figure 4A. The blue curve represents the change of divergence nodes number based on Figure 4B.

The divergence time based on the two datasets used for time calibration is consistent with previous genome-based analysis results (Figure 4; Wang et al., 2016). Three of the “putative new species,” *Mesocestoides* sp. RKZ08, *Mosgovoyia* sp. SQ20, and *Hydatigera* sp. XHPW10, might have originated from a similar phase in the late Miocene, while *Paranoplocephala* sp. RKZ13 diverged during the Pleistocene (Figure 4). By summarizing the number of divergent time nodes in the divergence time trees over time, it was found that the trees of *mt* PCGs and *cox*1 + *nad*1 produced similar differentiation rate trends: there was a slight acceleration of the evolutionary rate in the period 14–24 Mya, and a marked acceleration during the period 4–10 Mya (Figure 5). However, compared with *cox*1 + *nad*1, the differentiation rate of *mt* PCGs was faster in the 14–24 Mya but slower in 4–10 Mya, which may have been caused by the species bias used in both datasets.

## Discussion

Given the rich diversity and the large rodent population in western China and the few published reports of tapeworms in rodents, except for Taeniidae (Xiao et al., 2005; Zhao et al., 2014; Wang et al., 2018; Zhang et al., 2018; Wu et al., 2021), the present knowledge of tapeworm biodiversity in rodents in western China suggests far greater biodiversity yet to be uncovered.

Here, the *mt* genes and 18S or 28S rDNA fragments of four (two larvae and two adults) unidentified Cyclophyllidea species differed from their related species (Supplementary Table 10). However, due to the specimen distortion and insufficient specimen encountered in this study, it is not clear whether they have been described morphologically. All four species showed apparent discordance percentage identity with the related species in their *mt*DNA and 18S or 28S rDNA, which is the common DMD pattern across the animal kingdom, and demonstrated in *Echinococcus granulosus sensu stricto* (Kinkar et al., 2017), possibly due to the parthenogenetic inheritance of mitochondria, gene flow and recombination in the nuclear genome (Toews and Brelsford, 2012).

The larvae *Hydatigera* sp. XHPW10 was found to live in the livers of both zokors from northeast of QTP and plateau pika from southwest of QTP (Supplementary Table 1). This adds to the recently described cysticerci species in plateau pika from Qinghai province (Wu et al., 2021). Here the *T. caixuepengi* larva was also found to be parasitic in plateau pikas from Xietongmen, Saga and Sa’gya county of Tibet and Qilian county of Qinghai (Supplementary Table 1), and so far, not found in other sympatric rodent species. Due to the absence of comparative studies, it is currently not clear if this species has preference only for plateau pikas as its intermediate host. The larvae of *T. crassiceps* and *V. mustelae* were, respectively, found in Jeminay and Xinyuan county of Xinjiang autonomous region (Supplementary Table 1), which were also distributed on the northeast QTP (Li et al., 2013; Zhao et al., 2014). Wide geographic distributions of identical species suggest that their endemic geographic range should be far beyond the available survey data. Except for *Mesocestoides* sp. RKZ08 identified in this study, the *Me. litteratus* is another species of the genus *Mesocestoides* reported in Qinghai and Heilongjiang provinces of China (Wang et al., 2006; Li et al., 2013). However, there is no previous record of *Paranoplocephala* spp. and *Mosgovoyia* spp. available in China except for *Paranoplocephala* sp. RKZ13 and *Mosgovoyia* sp. SQ20 found in this study.

Complete *mt* genomes of the four “putative new species” were sequenced and annotated, and the sequences were clearly different from all available *mt* genomes sequences; however, they were similar in length, gene order and composition as other cyclophyllideans with respect to rRNA, tRNA, and protein-encoding genes (Le et al., 2000; Jeon et al., 2005, 2007; Wu et al., 2021). Different arrangement of genes occurred in tRNA genes between *nad*5 and *nad*6 genes, but was consistent with their respective most relative species, matching the expectation that the arrangement of *mt* genes partly determines the genetic relationship of parasites (Gazi et al., 2016). Moreover, repeat copies of tRNA gene between the *nad*5 and *nad*6 genes in the *mt* genome of *E. granulosus* sequenced by third-generation sequencing have been reported (Kinkar et al., 2019). We also observed a repeat sequence of *trn*L and *trn*R genes between *nad*5 and *nad*6 genes of the *Paranoplocephala* sp. RKZ13 *mt* genome (Figure 3 and Table 1), which indicates that there may be hidden tRNA gene repeats in the *mt* genome of tapeworms that are hard to identify due to errors in PCR amplification and Sanger sequencing, techniques which often fail to recover repeat regions (Kinkar et al., 2019).

Genetic drift and adaptive differentiation between allopatric populations is responsible for most speciation amongst plants and animals (Turelli et al., 2001). For parasites, however, host association is a key driver in their evolution. Host switching among sympatric populations may lead to ecological isolation, so sympatric speciation of parasites is common (de Meeûs et al., 1998; Paul, 2002; Huyse et al., 2005; Wu et al., 2021). These two models of evolution are not in conflict: the adaptation of parasite to specific host is like the adaptation of animal to specific living environment; so, the evolution of the host, especially its immune system, may be viewed as equivalent to the change of the living environment for the parasite. The environmental and climatic changes caused by the uplift of the QTP are a major driving force for the evolution of associated biotas (Favre et al., 2015). Paleobotanical data suggest that the southeastern margin of the QTP was dominated by a warm and humid subtropical or tropical climate during the Miocene due to the influence of South Asian and East Asian monsoons (Sun and Wang, 2005; Jacques et al., 2011). Since the mid-Miocene, the significant rise of the Himalayas and the Tianshan Mountains, together with worldwide cooling, incurred dramatic changes in air circulation, leading to gradual aridification of the QTP and Central Asia (Miao et al., 2012; Favre et al., 2015). Finally, in the Late Miocene and Early Pliocene, QTP uplift resulted in the accumulation of global ice and the eventual disappearance of the Tethys Sea, which also contributed to the drying of Central Asia (Lu and Guo, 2013).

These timelines of climatic and environmental changes caused by the uplift of the QTP are highly consistent with the timelines of the differentiation rate of Cyclophyllidea species analyzed in this study (Figures 4, 5) and that of plateau pika analyzed in Wang et al. (2020). We speculated in this study that the differentiation of cyclophyllideans may have been driven by host evolution caused by the uplift of the QTP. During the tropical period of QTP, the optimum living environment created the biological diversity (Cai et al., 2020; Päckert et al., 2020), and the species of Cyclophyllidea gradually differentiated. With the rapid uplift of the QTP, the environment changed into a dry and cold climate (Lu and Guo, 2013), and species differentiation of Cyclophyllidea was accelerated by the rapid adaptive evolution of their hosts and geographical isolation caused by the radiation of hosts to the Palaearctic (Favre et al., 2015; Xing and Ree, 2017; Päckert et al., 2020). Finally, in the last 2 million years, Cyclophyllidea differentiation demonstrated an accelerated diversification based on *cox*1 + *nad*1 divergence tree (Figure 5), which may be related to the evolution and broad distribution of mammals in Eurasia and the associated population expansion and migration of hominids from Africa to Asia (Dennell, 2004; Rohland et al., 2005; Brugal and Croitor, 2007; Turner et al., 2008; Klein, 2009; Terefe et al., 2014; Wang et al., 2016). The Host-parasite Database of Natural History Museum (HPDNHM) found that most tapeworm were prevalent mainly in the Palaearctic,^5^ which is also consistent with the viewpoint that the order Cyclophyllidea originated from the QTP. The Nearctic is another major endemic area where Cyclophyllidea species may have spread over land Bridges across the Bering Strait. However, some species parasitizing birds and other economic and companion animals tend to show a global epidemic, which may be due to the long-distance migration of birds and the spread of human trade and activities.

Phylogenetic reconstruction reveals that some classifications of Cyclophyllidea species may need to be redefined. Closely related species of tapeworm parasites often have similar host specificities and life history (Scholz et al., 2021), a pattern common in the HPDNHM and in our evolutionary analyses, and may provide a basis for revising the classification of Cyclophyllidea species; for example, the family Hymenolepididae and Anoplocephalidae can be divided into multiple families (Figures 1A–C), and *Thysanotaenia congolensis* should be reclassified into family Davaineidae (Figures 1A,C). However, considering that there is still a large number of undiscovered species, which may provide better support for classification, the current classification status is likely to remain for some time.

## Conclusion

In conclusion, this study expands the biodiversity of Cyclophyllidea in rodents in QTP and its surrounding mountain systems, and suggests an “out of QTP” hypothesis for the Cyclophyllidea, wherein species differentiation was driven by the uplift of the QTP. Although beyond the scope of this study to consider the evolutionary relationships and history of the whole cyclophyllideans, the species analyzed represent 10 of all 16 families, making this the most extensive study of the evolution of Cyclophyllidea order to date. Verifying the taxonomic revision and the “out of QTP” hypothesis requires more sampling and investigation, including data on a wider geographic and host range, and molecular studies uncovering patterns of host-parasite co-evolution.

## Data Availability Statement

The datasets presented in this study can be found in online repositories. The names of the repository/repositories and accession number(s) can be found below: https://www.ncbi.nlm.nih.gov/genbank/, MZ476188–MZ476193; https://www.ncbi. nlm.nih.gov/genbank/, MW808979–MW808982; https://www. ncbi.nlm.nih.gov/genbank/, OM140661–OM140665.

## Ethics Statement

The animal study was reviewed and approved by the Animal Ethics Procedures and Guidelines of the People’s Republic of China. Animal Ethics Committee of Lanzhou Veterinary Research Institute, Chinese Academy of Agricultural Sciences (No. LVRIAEC2012-007).

## Author Contributions

Y-DW, LL, H-BY, and W-ZJ conceived and designed the experiments. Y-DW, G-DD, L-SZ, A-MG, Y-TW, and YF conducted the sample collection. Y-DW, G-DD, X-WN, and NS performed the experiments. Y-DW and G-DD performed the data analyses. Y-DW prepared the figures and wrote the manuscript. DTJL, JO, W-HL, N-ZZ, and B-QF provided very constructive suggestions for revisions. All authors read and approved the final manuscript.

## Conflict of Interest

The authors declare that the research was conducted in the absence of any commercial or financial relationships that could be construed as a potential conflict of interest.

## Publisher’s Note

All claims expressed in this article are solely those of the authors and do not necessarily represent those of their affiliated organizations, or those of the publisher, the editors and the reviewers. Any product that may be evaluated in this article, or claim that may be made by its manufacturer, is not guaranteed or endorsed by the publisher.

## Footnotes

1
https://www.ncbi.nlm.nih.gov/taxonomy

2
https://blast.ncbi.nlm.nih.gov/Blast.cgi

3
https://chlorobox.mpimp-golm.mpg.de/geseq.html

4
http://130.235.46.10/ARWEN/

5
https://www.nhm.ac.uk/research-curation/scientific-resources/taxonomy-systematics/host-parasites/database/index.jsp

## Supplementary Material

Supplementary Figure 1 Maximum likelihood analysis based on the 28S rDNA fragments of classified cyclophyllideans in NCBI Taxonomy Database. The ▲ after the species name represents “putative new species”. The outgroup is the same as Figure 1. Bootstrap frequency support values are stated only for nodes where > 80%.
