# Supplementary figures and images for: Expansion of Cyclophyllidea Biodiversity in Rodents of Qinghai-Tibet Plateau and the “Out of Qinghai-Tibet Plateau” Hypothesis of Cyclophyllideans
Source: Front Microbiol. 2022 Feb 8;13:747484. doi: 10.3389/fmicb.2022.747484 (PMC8861457; doi:10.3389/fmicb.2022.747484)

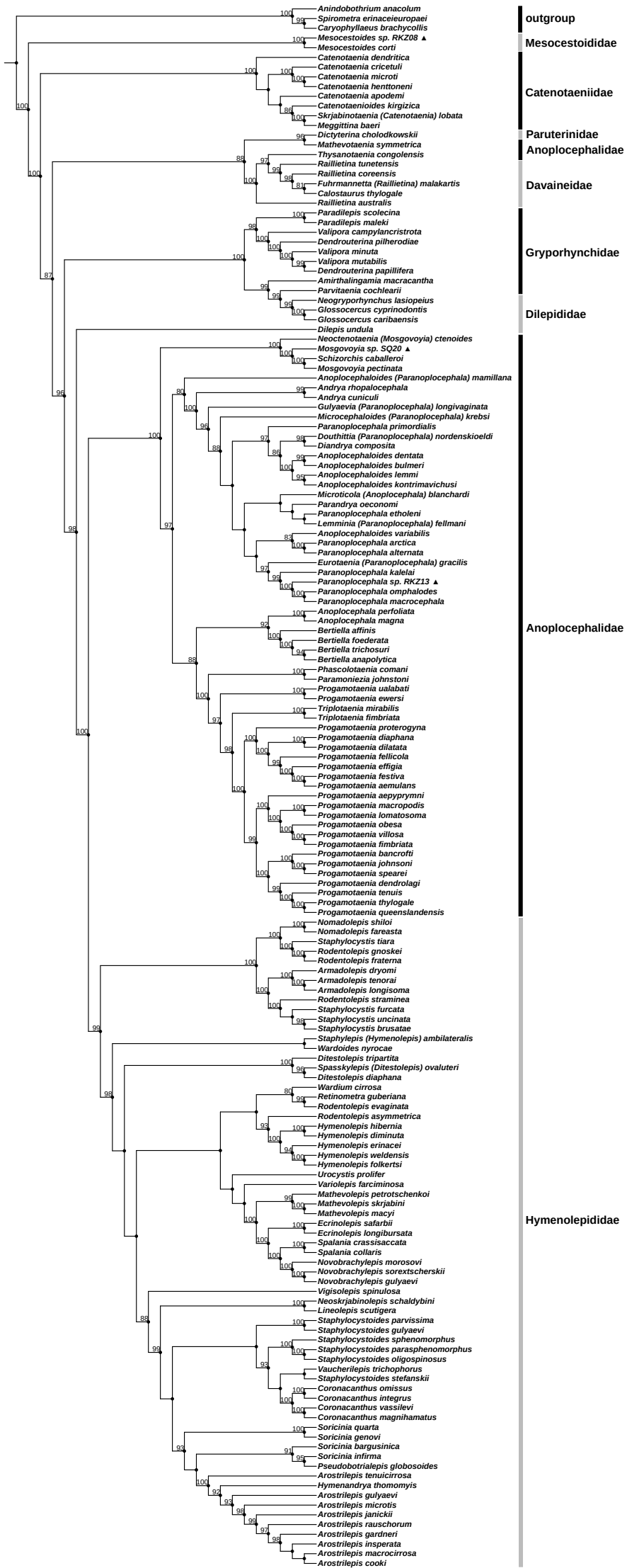

Supplement: Supplementary Figure 1 — Maximum likelihood analysis based on the 28S rDNA fragments of classified cyclophyllideans in NCBI Taxonomy Database. The ▲ after the species name represents “putative new species”. The outgroup is the same as Figure 1. Bootstrap frequency support values are stated only for nodes where > 80%. [file Data_Sheet_1.PDF]
